# Hazard Perception in Older Drivers With Eye Disease

**DOI:** 10.1167/tvst.10.1.31

**Published:** 2021-01-22

**Authors:** Joanne M. Wood, Alex A. Black, Kaarin J. Anstey, Mark S. Horswill

**Affiliations:** 1 Centre for Vision and Eye Research, School of Optometry and Vision Science, Queensland University of Technology, Brisbane, Australia; 2 School of Psychology, University of New South Wales, Sydney, Australia; 3 Neuroscience Research Australia, Sydney, Australia; 4 UNSW Ageing Futures Institute, University of New South Wales, Sydney, Australia; 5 School of Psychology, The University of Queensland, Brisbane, Australia

**Keywords:** older drivers, eye disease, hazard perception, driving safety, visual impairment

## Abstract

**Purpose:**

Timely detection of hazards is a key driving skill; however, the hazard perception of drivers with eye disease and related visual changes and the visual predictors of hazard perception are poorly understood.

**Methods:**

Participants included drivers aged 65 years and older with a range of eye diseases, including cataract, age-related maculopathy (AMD), and glaucoma (*n* = 99; mean age, 75.4 ± 6.4 years) and controls (*n* = 118; mean age, 72.2 ± 5.5 years). Visual performance was assessed using clinical measures (visual acuity, contrast sensitivity, visual fields) and non-clinical measures (useful field of view, motion sensitivity). Participants completed a computer-based hazard perception test (HPT) that has been related to driving performance and crash risk.

**Results:**

Participants with eye disease exhibited a 0.73-second delay in HPT response times compared to controls (6.61 ± 1.62 seconds vs. 5.88 ± 1.38 seconds; age-adjusted *P* = 0.012). Participants with glaucoma exhibited significantly delayed responses compared to those with AMD (*P* = 0.038) and controls (*P* = 0.004). Poorer motion sensitivity (standardized β = 0.27; *P* < 0.001), visual acuity (β = 0.21; *P* = 0.002), and better-eye mean defect (β = –0.17; *P* = 0.009) were most strongly associated with delayed HPT responses. Motion sensitivity remained significantly associated with HPT responses, adjusted for visual acuity and visual fields.

**Conclusions:**

HPT responses of older drivers with eye disease were delayed compared to controls and translate to an estimated 16-meter longer stopping distance when traveling at 80 km/hr. Decreased motion sensitivity was most strongly associated with delayed HPT responses.

**Translational Relevance:**

HPT tests can provide insight into difficulties regarding road hazard detection of older drivers with eye disease and provide a potential avenue for interventions to improve road safety.

## Introduction

Older drivers with eye disease and visual impairment have reduced driving ability and safety compared to those with normal vision, as assessed using a range of outcome measures.^1^ One important component of driving that is likely to be impacted by visual impairment is hazard perception, which is the ability to anticipate potential road hazards in order to avoid a collision.^2^

On-road studies support the suggestion that drivers with eye disease and associated visual impairment have reduced hazard perception. One study showed that drivers with age-related macular degeneration (AMD) made more observational errors than controls,^3^ whereas drivers with binocular field loss (primarily from glaucoma) had impaired anticipation skills.^4^ In another study, drivers with mild to moderate glaucomatous loss were more likely to receive a driver instructor intervention that was related to difficulties with detection of peripheral obstacles, hazards, and unexpected events, compared to controls.^5^

Assessment of drivers’ hazard perception ability during open-road assessments, however, remains a challenge, given that the number and nature of potential hazards vary due to differences in traffic conditions from one assessment to another. A useful alternative is to assess hazard perception ability under controlled laboratory-based conditions, an approach that has been commonly used in driving research.^6^^–^^10^ This approach involves presentation of a series of video clips involving real traffic conditions that contain a range of traffic hazards, where hazard perception is defined as the ability to anticipate and respond to potentially dangerous situations on the road.^7^

Studies of younger drivers have reported that delayed hazard perception test (HPT) response times are associated with crash involvement in both retrospective^11^^,^^12^ and prospective studies,^13^ as well as with increased frequency of heavy braking events during real-world driving.^14^ One study also showed that delayed HPT times were associated with increased self-reported crash involvement in a retrospective study of older drivers.^15^ However, these studies have been undertaken in general driving populations and not specifically in drivers with visual impairment related to eye disease, and the HPT response times of drivers with visual impairment have been reported in only a few studies. Most have explored the effects of simulated visual impairment on HPT response times using repeated-measures designs in individuals with normal vision, where the effects of optical blur (+2.00 diopter sphere [DS]),^10^ moderate levels of simulated cataracts,^16^ and simulated visual field loss^17^^,^^18^ resulted in slowing of HPT response times relative to performance with normal vision. Interestingly, although field loss in the superior field had a greater impact on HPT times than in the inferior field,^17^ whether or not field loss was right or left of fixation did not differentially impact HPT times.^18^ Only one study has explored HPT performance in those with true visual impairment related to eye disease, demonstrating that older drivers with glaucoma had slower HPT times than age-matched controls.^19^

The visual predictors of hazard detection ability in drivers have also only been explored in a limited number of studies. In two studies of participants with normal vision, motion perception (coherence thresholds and drifting Gabor patches) was found to be the best predictor of HPT performance.^20^^,^^21^ The only study that has explored this relationship in a sample that included drivers with normal vision and drivers with glaucoma demonstrated that hazard perception ability was best predicted by motion sensitivity, the useful field of view, and worse-eye mean defect (MD).^19^

The primary aim of the current study was to compare the hazard perception performance of older drivers with a range of eye diseases with that of age-similar controls. We hypothesized that those with eye disease would have delayed HPT response times compared to those with normal vision. A secondary aim was to explore the associations between visual function measures and hazard perception performance for older drivers with and without eye disease.

## Materials and Methods

### Participants

Participants included 217 regular drivers who were currently licensed in Australia and were 65 years of age and older, with or without eye disease, and who were part of two larger older driver studies. Participants were excluded if they had Parkinson's disease, a history of dizziness or vestibular disease, used a walking aid, or had cognitive impairment (Mini-Mental State Examination score < 24 of 30).^22^ Participants with eye disease were recruited from the clinical records of the Queensland University of Technology (QUT) Optometry Clinic and private ophthalmology practices in South-East Queensland and were diagnosed as having a pre-existing eye disease. The age-similar control participants were recruited as a convenience sample from our existing database of volunteers with no eye disease, as well as from the QUT optometry clinic and newspaper advertisements.

The study followed the tenets of the Declaration of Helsinki and was approved by the QUT Human Research Ethics Committee. Participants were given a full explanation of the study, experimental procedures, and possible consequences, and written informed consent was obtained.

### Visual Assessment and Driving Characteristics

All participants underwent a comprehensive eye examination that included ophthalmoscopy and slit-lamp biomicroscopy to confirm their eligibility for the study. Participants also completed a battery of visual tests, which were conducted binocularly while the participants wore their habitual distance correction.

#### Visual Acuity

Distance high-contrast visual acuity was measured with the Early Treatment for Diabetic Retinopathy Study chart at 5 meters at a luminance of 100 cd/m^2^, using the letter-by-letter scoring method.^23^

#### Contrast Sensitivity

Letter contrast sensitivity was measured using the Pelli–Robson Contrast Sensitivity chart at 1 meter at a luminance of 110 cd/m^2^, using the letter-by-letter scoring method.^24^ A +1.00 DS lens was used to compensate for the working distance.

#### Visual Fields

Monocular visual fields were assessed in each eye using the SITA-Standard 24-2 threshold strategy on a Humphrey Field Analyzer Model 750 (Carl Zeiss Meditec, Dublin, CA). Binocular visual fields were also measured using the binocular Esterman test with participants wearing their habitual driving spectacles, if any, as is recommended; the Esterman efficiency score (percentage of points seen) was recorded.

#### Motion Sensitivity

Central motion sensitivity was measured using a computer-generated random-dot kinetogram at a working distance of 3 meters.^25^^,^^26^ A field of dots presented within a smaller central patch of dots moved in one of four directions (up, down, left, or right) over four discrete steps (total stimulus duration 400 ms). Participants were instructed to report the direction in which the central dots were perceived to be moving. Pixel displacement between frames was varied in a two-down, one-up staircase, with eight reversals; thresholds were given as the minimum displacement threshold (log deg arc).

#### Useful Field of View

The computer-based Useful Field of View (UFOV) Version 6.0.8 (Visual Awareness Research Group, Punta Gorda, FL) was used to measure visual processing speed and divided attention (subtest 2). This test has been shown to have high reliability and validity in the prediction of driving ability and crash risk in older adults.^27^^,^^28^

#### Hazard Perception Test

Hazard perception was measured using a computer-based measure of hazard perception,^15^^,^^29^ similar to that used in Australia and the United Kingdom for driver licensing. Participants viewed 20 video clips of real traffic situations which included potential traffic conflicts, filmed from the driver's perspective. Traffic conflicts were defined as situations in which the camera car would hit another road user if no evasive action was taken. Participants were asked to tap the computer monitor as early as possible to indicate the location of road users who were likely to be involved in traffic conflicts with the camera car. Participants wore their habitual computer or near-vision spectacles that enabled them to view the computer screen at a viewing distance of between 40 and 60 cm. The primary outcome measure of the HPT was the response time to selected traffic conflicts, measured from the first point the road user involved in the conflict could be identified to that time when the participant responded. The raw response times to each traffic conflict were converted to *z*-scores, using the means and standard deviations (SDs) of all responses in the current sample to each given conflict, in order to standardize responses. The *z*-scores were averaged across all clips and converted back into a response time (seconds) using the means and SDs of responses averaged across all participants to aid in the interpretation and reporting of the results.^7^^,^^10^

### Analysis

Statistical analyses were performed using SPSS Statistics 25.0 (IBM Corporation, Armonk, NY), and the level of significance was set at *P* < 0.05. Descriptive statistics were used to analyze the demographic, vision, and hazard response data.

Group differences in vision function characteristics were analyzed with independent sample *t*-tests. Linear regression models were used to compare group differences in HPT performance, adjusted for age. Linear regression models controlling for age were also used to explore the associations between each of the vision measures and hazard response time separately. To investigate the independent associations between the vision measures and HPT performance, a backward linear regression model was conducted using the significant visual predictors and controlling for age.

Data were checked to ensure that the statistical test assumptions were met, and goodness-of-fit tests for the regression models were evaluated. The collinearity diagnostics were checked to ensure that there was no undue bias in the backward linear regression analyses due to multicollinearity.

## Results

The sample consisted of 217 licensed drivers, including 99 with eye diseases (mean age, 75.4 ± 6.4 years) and a control group of 118 visually normal drivers (mean age, 72.2 ± 5.5 years). The drivers with eye disease had a range of conditions, including cataracts (*n* = 25); glaucoma (*n* = 22); AMD (n = 28); other retinal conditions, including diabetic retinopathy (*n* = 8); neurological conditions, including stroke (*n* = 4); and other visual conditions (*n* = 12). The participants’ demographic and visual function characteristics are presented in Table 1. The group with eye disease were significantly older than those with normal vision (by 3.2 years; *P* < 0.001), but there was no significant difference in sex distribution. All measures of visual function were significantly worse in the group with eye disease compared with the controls, including visual acuity, contrast sensitivity, visual fields, motion sensitivity, and speed of visual processing (UFOV).

**Table 1.** Demographic and Visual Function Characteristics of Participants with Eye Disease and Visually Normal Controls

	Eye Disease (*n* = 99)	Controls (*n* = 118)	*P* ^*^
Demographic			
Age (y), mean (SD)	75.4 (6.4)	72.2 (5.5)	<0.001
Female sex, *n* (%)	32 (32)	44 (37)	0.48
Vision, mean (SD)			
Binocular visual acuity (logMAR)	0.03 (0.13)	–0.06 (0.10)	<0.001
Binocular contrast sensitivity (log units)	1.69 (0.15)	1.80 (0.02)	<0.001
Visual field MD, better eye (dB)	–1.69 (3.09)	0.25 (1.44)	<0.001
Visual field MD, worse eye (dB)	–5.21 (6.57)	–0.68 (1.67)	<0.001
Esterman efficiency score (% points seen)	95.28 (6.49)	97.31 (3.52)	0.004
Motion sensitivity (log deg arc)	–1.57 (0.25)	–1.80 (0.15)	<0.001
UFOV subtest 2 (ms)	214.3 (155.9)	131.7 (123.7)	<0.001

^*^Independent samples *t*-test used for continuous variables and χ^2^ test for categorical variables.

Participants with eye disease exhibited a 0.73-second slowing of hazard response times compared to controls (6.61 ± 1.62 seconds vs. 5.88 ± 1.38 seconds), which was significant in the age-adjusted analyses (*P* = 0.012). The HPT performance of the larger eye disease subgroups (cataracts, glaucoma, and AMD) compared with the visually normal controls (Table 2, Fig.) demonstrated a significant effect of group, even when adjusted for age (χ^2^ = 9.17; *P* = 0.027). In the pair-wise comparisons with the control group, all eye disease groups exhibited slower HPT times, but only the glaucoma group was significantly worse (*P* = 0.004). There were also differences in HPT times across the three eye conditions, but only the glaucoma group was significantly worse than the AMD group (*P* = 0.038), but no other significant differences were found (*P* > 0.21).

**Table 2.** Hazard Response Time As a Function of Group

	Mean ± SD			
	Control (*n* = 118)	Cataract (*n* = 25)	AMD (*n* = 28)	Glaucoma (*n* = 22)
Hazard response times (s)	5.88 ± 1.38	6.39 ± 1.49	6.32 ± 1.41	7.07 ± 2.01

**Figure.**
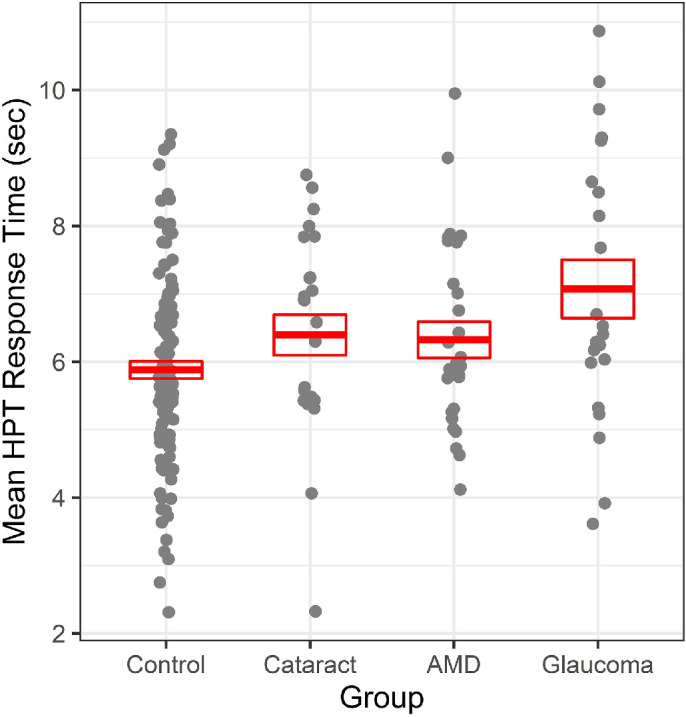
Hazard perception response times as a function of group, with *red lines* and *boxes* representing means and standard errors.

In age-adjusted analyses, delayed HPT response times were most strongly associated with poorer motion sensitivity (standardized β = 0.27; *P* < 0.001), visual acuity (standardized β = 0.21; *P* = 0.002), and better eye MD (standardized β = –0.17; *P* = 0.009) and worse eye MD (standardized β = –0.15; *P* = 0.022). The other vision measures were not significantly associated with hazard response times in the age-adjusted analyses.

A backward multiple regression corrected for age was conducted to determine the best independent predictors of HPT response times. The final model was highly significant (*F*_3,213_ = 14.4; *P* < 0.001; *R*^2^ = 17%) and included motion sensitivity (standardized β = 0.24; *P* = 0.001), age (standardized β = 0.19; *P* = 0.008), and better eye MD (standardized β = –0.11; *P* = 0.089).

## Discussion

The findings demonstrate that HPT response times were significantly slower in participants with eye disease, even when adjusted for age, and participants with glaucoma had the slowest HPT responses times. Importantly, motion sensitivity was most strongly associated with HPT response times, even when adjusted for age and the other vision measures.

In this study, HPT responses times were more delayed in older drivers with eye disease, by 0.73 second, compared to controls. This is consistent with previous studies showing delays in response times for older drivers with glaucoma compared to age-matched controls,^19^ and in a younger population with simulated cataracts.^16^ Importantly, these findings extend the results of previous studies by including a large range of older drivers with various eye diseases, including cataracts, glaucoma, and AMD. These findings provide evidence that the ability to predict safety-relevant objects within everyday traffic scenes is reduced in those with eye disease and may contribute to the increased crash risk and reduced driving ability reported in these groups. The between-group difference in HPT response times would translate to an estimated 16-meter longer stopping distance for a driver traveling at 80 km/hr^30^ due to delayed response and initiation of braking actions, which could have important consequences in terms of collisions with other vehicles, pedestrians, or cyclists.

Interestingly, the participants with glaucoma demonstrated the slowest response times of all of the participants. The finding of slowed HPT response times in older adults with glaucoma concurs with a previous study.^19^ In the present study, drivers with cataracts did not exhibit any significant slowing of hazard response times relative to controls. In contrast, simulated cataracts have been reported to slow hazard response times,^16^ but only for a cataract simulation that reduced contrast sensitivity to much lower levels than those found here for true eye disease from cataracts. Importantly, the drivers with cataracts in the present study had time to adapt to their eye disease, as cataracts typically develop over a period of years, in contrast to studies that use simulated cataracts that provide minimal adaptation time.

Of the vision measures, motion sensitivity exhibited the strongest association with HPT response times across all participants, even when adjusted for age and other visual functions, including standardized measures of visual acuity and visual fields. This suggests that detection of small amounts of motion is an important factor underlying the capacity to detect and predict road hazards within the driving environment. This finding is important given that motion sensitivity is impaired in a range of eye diseases, including glaucoma^31^ and AMD.^3^ It also aligns with reports by other studies that motion sensitivity is associated with HPT response times in those with glaucoma^19^ and with on-road driver performance in older adults with and without eye disease.^25^^,^^26^ This association is likely to arise because the driving environment is dynamic, due to the motion of the driver's vehicle and that of other road users who may become road hazards. Drivers need to be able to detect the speed and direction of motion of safety-relevant elements in the road environment, and the ability to do this is captured by the central motion sensitivity task.

UFOV processing speeds have been previously shown to be significantly associated with HPT response times for drivers with glaucoma and visually normal controls;^19^ however, these associations failed to reach significance when adjusted for age in the current study, in both the full sample and the subsample of glaucoma participants. This lack of association in the current study may be a result of a smaller sample size, as well as variations in the location of the field defects in the glaucoma participants between studies.

It is important to consider the findings of this study in light of its relative strengths and weaknesses. Strengths include the assessment of licensed drivers with a range of visual characteristics measured using a comprehensive vision testing battery. The participants had commonly occurring age-related eye diseases, such as cataract, glaucoma, and AMD, and their hazard perception responses were tested under controlled conditions. However, as with simulator-based studies, although the stimuli were realistic, in the sense of using video-based footage of genuine driving conflicts, they are not perceptually identical to those encountered in real-world driving. For example, by virtue of the constraints of the dynamic range of the monitor, the video stimuli had lower levels of luminance than would be experienced under normal daytime conditions, particularly on sunny days, and the visual angle of the presentation was limited by the computer monitor size and thus is not representative of the true driving environment.

In summary, this study demonstrated that HPT response times are slowed in older drivers with a range of common eye diseases that are typically found in the driving population and that motion sensitivity is associated with these differences. Importantly, slower HPT responses have been shown to be associated with increased crash risk and poorer driving performance in both young and older drivers.^11^^–^^15^ In addition, hazard perception ability has been shown to be amenable to training designed to enable earlier prediction of potentially dangerous traffic situations. This type of training involves video-based exercises where, for example, drivers are asked to generate a verbal commentary regarding traffic video clips, noting what they are monitoring, hazards present, and predictions of what might happen next. Training also includes an expert driver commenting on the same clips, providing insight into how they could improve their road awareness and anticipation strategies. It has been demonstrated that a novel training program involving these types of exercises improved response times in older drivers in general by 0.81 seconds compared to baseline.^32^ This suggests a potential avenue for interventions in drivers with eye disease, which should be explored in future studies. Future work should also explore whether improvements in hazard perception response times translate to improved driving safety under real-world driving conditions.
